# The impact of phenotypic heterogeneity on fungal pathogenicity and drug resistance

**DOI:** 10.1093/femsre/fuaf001

**Published:** 2025-01-14

**Authors:** Lukasz Kozubowski, Judith Berman

**Affiliations:** Eukaryotic Pathogens Innovation Center, Department of Genetics and Biochemistry, Clemson University, Clemson, SC, 29634, USA; Shmunis School of Biomedical and Cancer Research, The George S. Wise Faculty of Life Sciences, Tel Aviv University, Tel Aviv, 69978, Israel

**Keywords:** fungi, phenotypic heterogeneity, fungal pathogenesis, medical mycology, drug resistance, antifungal drugs

## Abstract

Phenotypic heterogeneity in genetically clonal populations facilitates cellular adaptation to adverse environmental conditions while enabling a return to the basal physiological state. It also plays a crucial role in pathogenicity and the acquisition of drug resistance in unicellular organisms and cancer cells, yet the exact contributing factors remain elusive. In this review, we outline the current state of understanding concerning the contribution of phenotypic heterogeneity to fungal pathogenesis and antifungal drug resistance.

## Teaser

This review discusses the contribution of phenotypic heterogeneity in fungi to pathogenesis and antifungal drug resistance.

## Introduction

Human fungal infections pose a significant global threat as recently emphasized in the first publication of a fungal priority pathogen list by the World Health Organization (https://www.who.int/news/item/25-10-2022-who-releases-first-ever-list-of-health-threatening-fungi) (Parums 2022). Fungal diseases are an increasing threat to human health because of the growing number of individuals with compromised immunity and the small number of effective antifungal drugs with low toxicity (Krysan 2017, Fisher et al. 2022). Most fungi prefer to grow at moderate to low temperatures. With the rise in global temperatures, more fungal species are expected to acquire the capacity to colonize and/or infect warm-blooded animals including humans (Garcia-Solache and Casadevall 2010, Gnat et al. 2021, Nnadi and Carter 2021).

**Figure 1.**
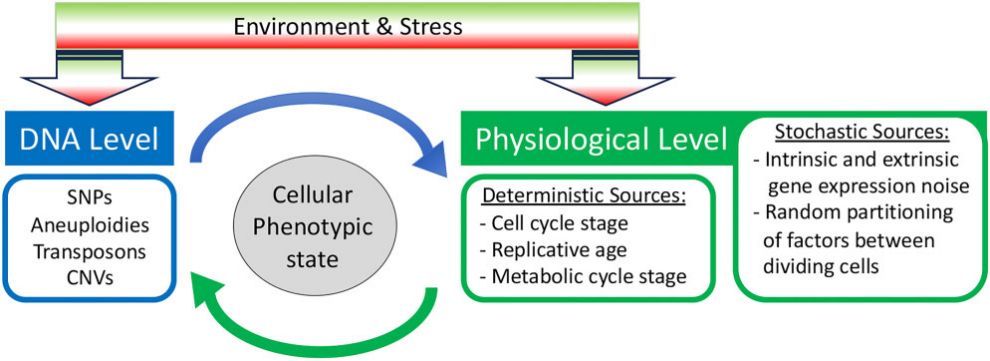
Genetic and non-genetic factors that drive phenotypic variability. Populations derived from a single progenitor cell can exhibit extensive phenotypic variability. Some arise through stable changes to cellular DNA [e.g. SNPs (single nucleotide polymorphisms), short tracts of nucleotide insertions or deletions, and transposons mobilized into or out of genes that influence growth)]. Others are unstable [e.g. local CNVs (copy number variations) and longer aneuploidies] and vary in their heritability in the absence of selection. Physiological (non-genetic) factors include deterministic sources and stochastic factors. Physiological and genetic factors affect one another: genetic background modulates the amplitude of variability caused by non-genetic factors; similarly, physiology can affect mutation, recombination, and chromosome missegregation rates. Congenic cells also may exist in a range of morphotypes (not shown in the schematic), providing another source of phenotypic heterogeneity.

Fungi harm their human hosts largely because they can circumvent both host immune defenses and antifungal therapies (Brown 2023), for example, by displaying an extensive variability in phenotype among clonally derived populations. While mutations or chromosomal aberrations that arise in a clonal population may contribute to phenotypic variability within the population, non-genetic sources can also cause such “phenotypic heterogeneity” (Fig. 1). Phenotypic heterogeneity has been documented in diverse microbes and has important ecological, industrial, and clinical implications (Ackermann 2015).

Recurrent infections, which appear to be cleared but return at a later time, present an important challenge to antifungal therapies (Perlin et al. 2017, Berman and Krysan 2020). Multiple factors contribute to the failure of current therapies to completely clear fungal infections (Brown 2023); the most prominent factor of these is the survival of some or all fungal cells within a population, despite the presence of the inhibitory drug. Specifically, *in vitro* assays that measure the minimum inhibitory concentration (MIC) of a population are assumed to indicate that if the drug is administered above the MIC, it should inhibit growth or kill the pathogen (Kohler et al. 2017, Perlin et al. 2017, Berman and Krysan 2020, Lee et al. 2021). However, if a proportion of the cells survive antifungal therapy, they may be sufficient to drive continued infection or reinfection. Thus, both genetic plasticity and phenotypic heterogeneity contribute to the establishment and further development of infections, by facilitating the ability of the pathogen to survive and grow in the face of antifungal treatments.

This review focuses on the effect of phenotypic heterogeneity on pathogenicity and drug responses in human fungal pathogens. Other recent reviews provide a broader perspective on the subject of fungal pathogenesis (Brown 2023), phenotypic heterogeneity in bacterial infections (Dewachter et al. 2019, Urbaniec et al. 2022), or in all microorganisms (Ackermann 2015, Zacchetti et al. 2018, Draghi 2019). Here, we first synthesize current knowledge regarding the sources and mechanisms of phenotypic heterogeneity as applied to microbial organisms in general, with an emphasis on those described in fungi. Subsequently, we then highlight aspects of phenotypic heterogeneity with clinical implications for fungal infections. Lastly, we discuss unresolved questions and possible future directions for studies on phenotypic heterogeneity in fungal pathogenesis.

## Sources and mechanisms of phenotypic heterogeneity

### Deterministic sources of phenotypic heterogeneity

Clonal cell populations usually are asynchronous, with individual cells having different cell division cycle stages, metabolic states, circadian rhythm responses, and replicative ages. All of these oscillatory behaviors can contribute to phenotypic heterogeneity (Fig. 1) (Sumner and Avery 2002, Tu et al. 2007). They can affect how individual cells respond to a drug or other stress, which can be detected, for example, by high-resolution microscopy. Furthermore, oscillations in the cell cycle stage, circadian rhythm state, and replicative age affect both gene expression patterns and metabolite fluxes, profoundly impacting cell physiology in all domains of life (Bell-Pedersen et al. 2005, Mellor 2016).

Connections between these oscillatory processes abound. Cell division cycle and metabolic oscillations have a period that depends on nutrient availability and other environmental conditions; circadian clock oscillations have less flexibility in period length, which is fixed to ~24 h (Bell-Pedersen et al. 2005, Tu et al. 2005, 2007). All three oscillatory behaviors coexist in cells and sometimes are coupled in response to environmental cues (Chen et al. 2004, Burnetti et al. 2016). While the impact of the cell division cycle on fungal pathogenesis has been studied extensively, the role of metabolic cycles and circadian rhythms remains largely unexplored during fungal infection (Ene et al. 2014, Perez-Martin et al. 2016, Costantini et al. 2020).

#### Cell division cycle

Cell division cycle asynchrony is a natural source of phenotypic heterogeneity (Lord and Wheals 1981). In both symmetrically and asymmetrically dividing cells, cell cycle stage affects the relative amount of DNA per cell, the degree to which transcription is active, the number of cytoplasmic components, and cell size. In *Saccharomyces cerevisiae*, each newly “born” (and relatively small) daughter cell initially differs from its mother cell in its size, cytoplasmic components, and gene expression patterns (Colman-Lerner et al. 2001, Aguilaniu et al. 2003, Henderson and Gottschling 2008). Whether there is a tight correlation between cell cycle stages at the transcriptional level remains unclear and may depend on a particular gene and environmental cues. For example, the environmental stress response in *Saccharomyces cerevisiae* is not cell cycle-dependent, yet stress exposure does lead to cell cycle-dependent modulations of ribosomal proteins (Gasch et al. 2017). Recent single-cell analysis of a *Candida albicans* laboratory strain responding to antifungal drugs found that cell populations in distinct cell cycle stages also expressed specific types of stress response genes (e.g. heat shock stress proteins during mitosis and oxidative stress genes during stationary phase) (Dumeaux et al. 2023), consistent with earlier reports on synchronized cells (Brauer et al. 2008, Chiu et al. 2011, Senn et al. 2012, Hossain et al. 2021).

An extreme example of cell cycle-related phenotypic heterogeneity is the quiescent state, in which a subset of cells in the population exit the cell cycle and stop proliferating in response to nutrient depletion, but can resume growth if returned to nutrient-replete medium (Gray et al. 2004, De Virgilio 2012, Alanio 2020, Opalek et al. 2023). Quiescence is particularly prominent in yeast batch cultures exhausted of nutrients as they approach stationary phase (Werner-Washburne et al. 1993, Sun and Gresham 2021). Quiescent cultures contain several cell types, with a range of chronological ages, that differ in their metabolic activities, their ability to repair DNA, and their stress resistance (Allen et al. 2006, Werner-Washburne et al. 2012).

#### Non-random partitioning of components between dividing cells

Cellular division should in principle involve doubling of cellular content followed by equal division of this content between resulting two cells. In most cases doubling and halving of the DNA takes place at every cellular division. However, the distribution of cellular components other than DNA may or may not be equal between dividing cells, which contributes to phenotypic heterogeneity (Birky 1983, Huh and Paulsson 2011a, Ouellet and Barral 2012).

Depending on whether the division results in two identical cells or cells that differ in size and cellular content, we differentiate between symmetric and asymmetric division, respectively. However, numerous studies indicate that, in reality, truly “symmetric” division of all cellular content does not exist for any cell type at least for two reasons: (i) Many cytoplasmic components are subject to random distribution between dividing cells, which is an example of a stochastic source of phenotypic heterogeneity, discussed in the “Stochastic sources of phenotypic heterogeneity” section (Huh and Paulsson 2011b). (ii) Some components, especially those that are associated with the plasma membrane, are often subject to unequal distribution between dividing cells even in single-cell organisms that in principle are considered undergoing symmetric division (Bergmiller et al. 2017). This non-random distribution can be considered a deterministic source of phenotypic heterogeneity as it results from a difference between the “mother cell” (a cell that replicated the DNA) and the “daughter cell” (a cell that has received half of the replicated DNA from the mother). As illustrated in the following section, some of this heterogeneity has been associated with aging, whereas in other cases, the link to aging remains controversial.

##### Replicative aging

Replicative aging that contributes to phenotypic heterogeneity has been well documented in cells that divide asymmetrically, for instance, in stem cells or budding yeasts, including *Saccharomyces cerevisiae*, or pathogenic yeasts such as *Candida* species, and *Cryptococcus neoformans* (Henderson and Gottschling 2008, Bhattacharya et al. 2020, Manzano-Lopez and Monje-Casas 2020).

In yeasts, cellular components are preferentially retained in the mother cell such that daughter cells inherit a “fresh start” enabling them to undergo more divisions than their mother, leading mother cells to progressively accumulate components that contribute to aging. In this case, phenotypic heterogeneity is a natural consequence of differences in replicative cell age between newly born buds and older mother cells (Mortimer and Johnston 1959, Denoth-Lippuner et al. 2014).

Replicative age influences stress resistance: newly born daughters are more resistant to copper than their mothers (Sumner et al. 2003). Furthermore, genomic instability and the acquisition of mutations are affected by cell age (Knorre et al. 2018): for example, older *Saccharomyces cerevisiae* cells are more susceptible to the mutagen ethyl methanesulfonate (Kale and Jazwinski 1996). Conversely, resistance to UV mutagenesis exhibits a biphasic dependence on cell age, with “midlife” cells having the lowest susceptibility (Kale and Jazwinski 1996). Accordingly, different types of mutations may be acquired more frequently, depending upon cell replicative age.

Interestingly, asymmetric segregation and inheritance of potential aging factors have also been documented in symmetrically dividing unicellular organisms (Stewart et al. 2005). In both bacterial and yeast cells that divide symmetrically, a “mother” cell that replicates the DNA is marked by the old pole, whereas the daughter cell receives the new pole. Strikingly, proteins associated with the plasma membrane often preferentially segregate toward the old pole during division, resulting in mother–daughter asymmetry.

An example of phenotypic heterogeneity generated by unequal partitioning between dividing bacterial cells is the distribution of efflux pump proteins in *Escherichia coli*; such unequal partitioning may contribute to the downstream development of antibiotic resistance or tolerance by allowing a subpopulation of “older” cells with higher drug efflux levels to survive in the presence of the drug (Bergmiller et al. 2017).

Unequal partitioning of efflux pump proteins between dividing cells might also explain at least part of the antifungal tolerance phenotype. For instance, in more azole-tolerant isolates of *Candida albicans*, more cells in the population grow (Rosenberg et al. 2018) and a larger proportion of cells have lower intracellular drug levels, which could be due to increased efflux and/or decreased drug uptake.

Phenotypic heterogeneity due to the asymmetric partitioning of factors between yeast cells that divide symmetrically by fission has been documented in *Schizosaccharomyces pombe* (Nakaoka and Wakamoto 2017). The authors utilized time-lapse microscopy coupled with a microfluidic device and obtained single-cell lineage data to address the question of whether aging occurs in *Schizosaccharomyces pombe*. The authors documented preferential segregation of Hsp104-associated protein aggregates, which are a potential aging factor, in old-pole cell lineages. However, the authors found no evidence for those protein aggregates to contribute to cell death in *Schizosaccharomyces pombe* or to correlate to replicative aging under both favorable and stress conditions (Nakaoka and Wakamoto 2017). While this study indicates that aging in symmetrically dividing cells remains elusive, it does confirm that certain cellular constituents may partition preferentially with the old pole of symmetrically dividing cells, which may contribute to the deterministic form of phenotypic heterogeneity.

### Stochastic sources of phenotypic heterogeneity

Gene expression noise, which contributes to cell-to-cell variation in protein levels, may originate from inherent stochastic fluctuations in transcriptional activity, mRNA dynamics (both transcript production and stability), translation efficiency, and protein stability (Fig. 1) (Newman et al. 2006, Raj and van Oudenaarden 2008, Sanchez and Golding 2013, Liu et al. 2016). Gene expression noise can be extrinsic or intrinsic (Elowitz et al. 2002).

#### Extrinsic and intrinsic gene expression noise

Extrinsic noise is the noise that has a similar effect on all genes within a cell—for example, because the cell was exposed to some stress condition. Intrinsic noise is the inherent randomness in the expression of a specific gene; it originates from inconsistencies in the function of the transcriptional and/or translational machinery. A classic way to distinguish intrinsic and extrinsic noise is to compare the expression of two nearly identical genes, each expressing a different fluorescent reporter protein (Elowitz et al. 2002, Raser and O’Shea 2004). For example, two nearly identical, yet spectrally distinguishable, fluorescent proteins [e.g. cyan (CFP) and yellow (YFP)] can be expressed from an identical promoter in the same *E. coli* strain. Using this system, extrinsic noise is detected as a strong correlation in the expression of the two reporters. The inference is that the correlated expression of two genes in identical genomic contexts would be due to intracellular biochemical differences, such as differences in the numbers of RNA polymerases or ribosomes, which affect both genes similarly within a single cell (Elowitz et al. 2002).

By contrast, intrinsic noise is detected as expression that *differs* between the two fluorescent proteins in this same system (Elowitz et al. 2002). Intrinsic noise levels decrease as gene expression levels increase (Carey et al. 2013). Furthermore, intrinsic noise is inversely correlated with the number of molecules involved in regulating the expression of the gene (Elowitz et al. 2002). Thus, the more regulatory processes are involved in gene expression, the more likely that the gene exhibits intrinsic gene expression noise. Furthermore, even if hypothetically all extrinsic noise was eliminated (cells in the population contained equal numbers of factors essential for gene expression), the intrinsic noise in the expression of individual genes would remain (Elowitz et al. 2002).

#### Factors affecting gene expression noise

In bacteria, natural selection can reduce gene expression noise (Silander et al. 2012). In *Saccharomyces cerevisiae*, mechanisms that reduce gene expression noise and the resulting consequences on protein abundance have been documented for glucose metabolism genes as well as for genes involved in circadian rhythm regulation, among others (Vilar et al. 2002, Swain 2004, Metzger et al. 2015). By contrast, specific environmental conditions, such as stresses, increase gene expression noise. This suggests that phenotypic heterogeneity may promote adaptation to stresses that increase gene expression noise in yeasts (Blake et al. 2006, Fraser and Kaern 2009).

Rapid responses to stress often involve and require the TATA box: genes with a TATA box sequence upstream are far more likely to have high levels of gene expression noise and often encode genes that are upregulated in response to stress (Basehoar et al. 2004, Raser and O’Shea 2004, Blake et al. 2006). Furthermore, the relative binding of the TATA-box binding protein (TBP) to the TATA box sequence and other promoter elements modulate noise, which suggests a higher level of regulation (Ravarani et al. 2016). Other molecular factors that contribute to gene expression noise include nucleosome occupancy and histone modification patterns (Tirosh and Barkai 2008, Choi and Kim 2009, Hornung et al. 2012, Weinberger et al. 2012, Small et al. 2014), which are presumed to affect the accessibility of regulatory sequences important for gene expression.

The most significant factor predictive of gene expression noise in *Saccharomyces cerevisiae* was proposed to be transcription factor cooperation and competition for DNA binding sites (Parab et al. 2022). Given that several molecular mechanisms influence expression noise at the translational level, the fact that transcription factor interaction is found most predictive for noise at the level of protein synthesis is striking. One potential explanation is that the two datasets analyzed were derived from cultures grown at optimal, “non-stress” conditions (Newman et al. 2006, Nadal-Ribelles et al. 2019). This implies that transcription factor competition and cooperation for DNA binding in the absence of stress is the predominant predictor of gene expression noise. It also suggests that, under stress, expression noise in mRNA and protein levels is correlated (Parab et al. 2022).

#### Gene expression noise at the level of protein translation

Translational gene expression noise is seen in prokaryotes and eukaryotes. [L238] In *E. coli*, high glucose reduces intracellular pH, thereby slowing the peptide release from ribosomes and increasing stop codon readthrough. This yields a subset of proteins with extended C-termini, resulting in increased cell-to-cell heterogeneity of translated protein products encoded by a single gene (Zhang et al. 2020). Notably, cells with higher stop readthrough rates exhibit better recovery from acid stress, providing a link between phenotypic heterogeneity from translational readthrough and the ability to cope with environmental changes (Zhang et al. 2020).

Stop codon readthrough also has been described in mammals and fungi and may therefore constitute one of the conserved mechanisms connecting environmental stress to phenotypic heterogeneity at the level of translation (Stiebler et al. 2014). A dramatic example of connections between translational variability and stress responses comes from *Saccharomyces cerevisiae*, where the production and functionality of ribosomes depend on introns within ribosomal encoding genes (Warner 1999), which modulate the response to starvation (Parenteau et al. 2011, 2019). This intron-dependent bimodal expression of the *Saccharomyces cerevisiae* small ribosomal subunit Rps22B is modulated by osmotic stress and produces two subpopulations that differ in their sensitivity to osmotic stress (Lukacisin et al. 2022). Strikingly, two populations that differed in their levels of Rps22B expression exhibited reciprocal sensitivities to starvation, depending on the duration of growth under the low nutrient conditions (Lukacisin et al. 2022). When cells were placed in high glucose medium (higher than standard laboratory medium), they produced diverse levels of Rps22B as the culture approached stationary phase. This intron-dependent phenotypic heterogeneity is suggested to have evolved to accommodate a specific environmental niche: grapes rich in sugars (Lukacisin et al. 2022). In such a case, splicing-dependent regulation of specific ribosomal subunits may constitute a bet-hedging mechanism to diversify populations based on their ability to survive specific stresses.

Translational gene expression noise and its effect on antifungal drug responses also have been documented in pathogenic fungi, including *Candida albicans*, as described in detail in the “Effect of phenotypic heterogeneity on fungal pathogenesis and drug resistance” section.

#### Stochastic partitioning of cellular components between dividing cells

As discussed in the “Non-random partitioning of components between dividing cells” section, unequal partitioning of cellular components between dividing cells contributes to phenotypic heterogeneity. While for some cellular constituents, partitioning is dictated by the cell identity (and would be considered a deterministic source of phenotypic heterogeneity), for many cellular elements, especially those in the cytoplasm, including RNA molecules, proteins, and metabolites, partitioning is random (Huh and Paulsson 2011a). In this case, such a stochastic distribution may lead to relatively large differences between dividing cells, in particular when the overall abundance of those molecules is relatively low. Moreover, the apparent noise in molecule abundance observed among cells from a clonal population may be attributed to a composite of gene expression noise and stochastic partitioning of molecules during cellular division. Huh and Paulsson (2011a) performed mathematical modeling of protein abundance noise and proposed that much of the cell-to-cell heterogeneity that has been attributed to various aspects of gene expression noise instead comes from the stochastic distribution of molecules at cell division. The authors also devised quantitative methods to decouple the protein expression noise from the noise originating from the random partitioning of proteins between dividing cells (Huh and Paulsson 2011a).

## Effect of phenotypic heterogeneity on fungal pathogenesis and drug resistance

### Heterogeneity at the level of microbial colonies

Microbial colonies are striking examples of extensive phenotypic heterogeneity (Stewart and Franklin 2008) and a type of growth that is particularly important for studying the development of antimicrobial drug tolerance/resistance *in vitro* (described in more detail later). Microscopic monitoring of the growth of many single cells into many microcolonies facilitates estimations of the fraction of cells in the initial population that are tolerant/resistant or switch to the tolerant/resistant state. Typically, the term “microcolony” refers to cells that have grown on a semi-solid agar medium and have originated from a single cell.

#### Heterogeneity in colony growth rate

In bacteria, persister cells arrest growth or grow significantly slower than the remaining cells in the genetically clonal population and exhibit higher resistance or tolerance to antibiotics (Balaban et al. 2004, de Jong et al. 2011). In most cases, during drug exposure, persister cells are metabolically quiescent and they resume growth after the drug is removed from the culture (Prax and Bertram 2014). While most such mechanisms described in bacteria are based on bistable gene expression patterns resulting in a discrete bimodal distribution of growth rates, other reports found growth rate distributions in bacterial populations to be continuous (Levin-Reisman et al. 2010).

In fungal cells, growth rates for genetically clonal colonies vary. What underpins the variability in colony size in laboratory conditions (presumed to be non-stressed) and the increased phenotypic heterogeneity of colony sizes in the presence of stresses, including antifungal drugs, have been documented (Levy et al. 2012, van Dijk et al. 2015).

Using high-throughput microscopy to monitor the growth of thousands of *Saccharomyces cerevisiae* microcolonies, Levy et al. (2012) uncovered a continuous variation in colony growth rates within an isogenic population. This suggests that a complex combination of stochastic and deterministic factors contributes to the rate at which cells form microcolonies. By screening databases of genes involved in energy storage or mobilization genes (Newman et al. 2006, Brauer et al. 2008), they found that a trehalose metabolism gene, Tsl1, which encodes a cofactor subunit of trehalose synthase, exhibited expression variation that correlated with colony growth rate, survival of a heat shock, and the replicative age of cells. Consistent with these findings, the growth rate was heritable over several generations, implying that phenotypic heterogeneity in colony growth rate is largely, but not exclusively, driven by variation in the replicative age-dependent abundance of stress response proteins. They posit that this could be a common bet-hedging strategy in *Saccharomyces cerevisiae* and that it may be akin to phenomena documented in bacteria.

#### Heterogeneity within a single yeast colony

An additional level of complexity is due to phenotypic heterogeneity within a microcolony when it is grown on a semi-solid agar medium. The degree of intracolony phenotypic heterogeneity increases with increased colony size, as detected by vibrational spectroscopy (Choo-Smith et al. 2001). Differences in absorption, using Fourier transform infrared spectroscopy, revealed three layers within the colony (the outer layer, the middle layer, and the layer that is in contact with the medium surface) that differed significantly in spectral characteristics, and presumably represented the biochemical composition differences that are associated with intracolony phenotypic heterogeneity between cells in these regions (Choo-Smith et al. 2001).

Other studies described intracolony phenotypic heterogeneity resulting from metabolic variation between cells that form a microcolony. For example, subpopulations of *Saccharomyces cerevisiae* within the same colony, some that produce and others that consume certain amino acids, were analyzed using a proteomic approach termed “differential isotope labeling by amino acids” (DILAC) (Kamrad et al. 2023). The authors utilized a carbon labeling strategy that distinguished between lysine molecules obtained by synthesis versus uptake from the medium. To ask whether cells from a single colony are homogenous in partly producing and partly importing lysine or whether there were subpopulations of “importers” and “producers,” the authors analyzed specific peptides that contain exactly two lysine residues. The majority of peptides contained either both “heavy” or both “light” lysins, consistent with the presence of distinct producer and consumer subpopulations within the colony. This analysis was extended to define the proteome of “consumers” versus “producers,” both in bulk whole colony cell lysates as well as in separate fractions of cells representing the bottom and top parts of “young” colonies (grown for 48 h). The analysis uncovered subpopulations of fast growers that occupy the bottom of the colony and ferment and slower growers that are at the top of the colony and respire. Interestingly, cells from the “consumer” fraction were significantly more susceptible to amphotericin B, suggesting that the metabolic state of cells in different colony regions and/or their growth rate influences susceptibility to this fungicidal drug (Kamrad et al. 2023).

Similarly, heterogeneity resulting from the presence of auxotrophs within a community of yeast cells led to an increase in extracellular amino acid concentrations that stimulated metabolic flux and promoted tolerance to azole antifungals (Yu et al. 2022). The authors postulate that auxotrophs within populations promote increased intercellular metabolite exchange, effectively reducing intracellular drug concentrations. This allows metabolically interacting cells to grow slowly at drug concentrations above the MIC (Yu et al. 2022), a property of cells exhibiting antifungal drug tolerance. While heterogeneity was based on genetically distinct populations (prototrophs and auxotrophs) in this study, phenotypic heterogeneity (in genetically clonal populations) also may influence metabolic flux and therefore alter drug responses.

#### Phenotypic heterogeneity within colonies of filamentous fungi

Phenotypic heterogeneity within a colony of filamentous fungi requires special consideration (addressed by Zacchetti et al. 2018). For example, different parts of growing *Aspergillus niger* hyphae are specialized to optimize nutrient acquisition and to protect the fungus from unfavorable external conditions (Levin et al. 2007, Vinck et al. 2011). Fungal growth relies upon the secretion of enzymes that degrade organic material to release nutrients small enough to be reabsorbed by the fungus. These “exploring hyphae” of *A. niger* differentiate to express high levels of multiple secreted enzymes (Vinck et al. 2005, 2011). They also have high 18S rRNA content, suggesting that they are highly active in translation, whereas the other subpopulation of cells is less active (Vinck et al. 2011).

A significant factor responsible for phenotypic heterogeneity in filamentous colonies grown on a semi-solid substratum is the local composition of the medium occupied by different regions within the growing hyphae (Levin et al. 2007). By analyzing expression in five concentric zones of 7-day-old *A. niger* colonies grown on maltose or xylose, lower concentrations of nutrients were found within the central region relative to peripheral ones. Comparing the concentric zone transcriptome before and after the colonies were shifted to fresh carbon-containing medium, the authors could attribute half of the variation between concentric regions to media composition. What drives the remaining variation between the five concentric zones remains to be determined.

Relevant to antifungal therapy, phenotypic heterogeneity within filamentous colonies is seen in the response of hyphal compartments of *Aspergillus fumigatus* to treatment with caspofungin concentrations that elicit the paradoxical growth effect (Moreno-Velasquez et al. 2017). The paradoxical growth effect relates to cases where fungi are inhibited by a relatively low drug concentration but surprisingly grow at a concentration of a drug that is much higher (paradoxical concentration). *Aspergillus fumigatus* conidia developed into aberrant highly branched and thickened hyphae within 36 h of treatment with either inhibitory or paradoxical concentration of caspofungin. However, between 36 and 48 h, heterogeneity was observed in hyphae treated with the higher (paradoxical) concentration of the drug. Specifically, leading hyphae at the periphery of the microcolony treated with the higher concentration of the drug underwent a radical change in morphology from that of untreated hyphae, or hyphae treated with the low (inhibitory) concentration, indicative of a higher rate of apical extension. This hyphal heterogeneity in colonies treated with higher caspofungin concentrations correlated with dynamic localization of the caspofungin target, β-1,3-glucan synthase, Fks1. Whereas low and high concentrations of caspofungin resulted in aberrant localization of Fks1 to vacuoles (instead of typical localization to hyphal tips) in the first 36 h of treatments, only in samples treated with the high concentration for 48 h, the Fks1 localized to the hyphal tips in the morphologically distinct and faster growing leading edge of the colony (Moreno-Velasquez et al. 2017).

### Heterogeneity in fungal morphologies and their relationship to virulence

Fungal species commonly exhibit a range of morphological forms. Transitions between those morphological states can be relatively uniform, where almost all cells respond similarly to the external stimulus; alternatively, only a minority subpopulation of cells might respond to the stimulus. These two types of morphological responses may reflect a continuum of responsive states within a population of cells. We first will discuss the most important types of morphological variation in fungi, including those that affect entire populations, emphasizing the types of variability that affect pathogenicity. Subsequently, we will discuss specific examples of phenotypic heterogeneity that diversify genetically clonal populations into morphological subgroups that affect pathogenesis and antifungal drug responses.

#### Transitions between yeast and hyphae in dimorphic fungi

A striking example of morphological variation that is crucial for fungal pathogenicity is the thermal dimorphic transition, seen most dramatically in dimorphic pathogens such as *Histoplasma, Coccidioides*, and *Blastomyces* (Klein and Tebbets 2007, Jain and Fries 2009, Boyce and Andrianopoulos 2015, Van Dyke et al. 2019). With these pathogens, host body temperature (∼37°C) triggers the change from saprophytic, filamentous hyphae to the yeast form that infects humans (Beyhan and Sil 2019, McBride et al. 2019). Several factors associated with the yeast form contribute to the survival of dimorphic fungi in their host. For instance, *Histoplasma capsulatum* yeast cells, like the yeast of other dimorphic pathogens, can reside within macrophages to avoid interactions with the host immune system (Newman et al. 1990). Dimorphic yeast also can reside extracellularly, where the yeast-specific transcriptional program includes upregulation of genes that promote immune system evasion. For example, *Blastomyces* yeast cells upregulate Bad1, an adhesin that promotes adhesion to host tissues and evasion of the host immune response (Finkel-Jimenez et al. 2002). Of note, the yeast form of thermally dimorphic fungi is required for virulence and is the *only* morphology growing in the host (after conidial sporulation). Thus, the dimorphic switch is more deterministic than most of the phenotypic heterogeneity discussed here.

#### Transitions between yeast and hyphae in *Candida albicans*

*Candida albicans* is a commensal of mucosal tissues that can cause candidiasis; candidal infections can be either superficial or life-threatening systemic infections, most frequently seen in immunocompromised individuals. In contrast to the dimorphic pathogens discussed earlier, both the yeast and filamentous morphologies of *Candida albicans* coexist in the host and contribute to pathogenicity; yeast cells promote adhesion, bloodstream dissemination, and biofilm dispersal, and hyphae promote tissue invasion and damage, escape from host phagocytes and biofilm formation (Gow et al. 2002, Thompson et al. 2011). Thus, both yeast and hyphal morphologies are relevant to *Candida albicans* infection and may contribute to variability in the infecting population and in biofilm function.

Does infection by *Candida albicans* require the fungal population to exhibit morphological heterogeneity with proportions of both yeast and hyphal forms? And if so, in what ratio? A precise answer to this imprecise question is elusive, as the requirement for *Candida albicans* hyphae to cause disease may depend on the specific infection type. For instance, a yeast-locked mutant and a strain that grows exclusively as yeast in the host are both strongly attenuated in the murine tail vein model, underscoring the importance of hyphae for virulence in a bloodstream infection model of systemic candidiasis (Lo et al. 1997, Saville et al. 2003). However, while a strain with reduced expression of hyphal regulatory gene *EED1* is defective *in vitro* in damaging epithelial cells and macrophages and in a mouse model of intraperitoneal infection, it is virulent, especially at low infectious doses in the mouse tail vein model of systemic infection (Dunker et al. 2021). The authors hypothesize that the increased fitness of the mutant mediated by the better metabolic adaptation to the host environment has compensated for the deficiency in hyphae formation (Dunker et al. 2021). This suggests that the degree to which each of the morphological forms contributes to candidiasis depends on the type of host, the inoculum, and the route of entry (Dunker et al. 2021). Thus, in *Candida albicans*, the ratio of yeast to hyphal cells in a population may affect the progression and outcome of candidiasis; however, more precise quantification of the degree to which the phenotypic heterogeneity of a strain affects virulence remains to be determined.

#### Morphological heterogeneity at the colony level and its relationship to virulence

Fungi may display heterogeneity between colonies or within a single colony (termed “colony sectoring”), which often originates from a phenomenon termed “phenotypic switching.” Phenotypic switching refers to a relatively high frequency (higher than the somatic mutation rate) stochastic and reversible transitions between distinct cellular phenotypes/morphologies, occurring typically in a small fraction of the population (Jain et al. 2008). This contrasts with yeast to hyphae transitions, including dimorphism, which are triggered by specific environmental conditions and include all cells in a given population. In the context of microbial (micro) colony growth, phenotypic switching of cells within the colony (sectoring) appears when a phenotypic switch is propagated for several generations, after which cells can revert to the initial phenotypic state. Colony sectoring is readily identifiable, as the switch variant has unique characteristics resulting in a growth pattern that is distinct from the rest of the population. As switching between alternative phenotypic variants has been initially documented based on colony morphologies, we address this first in a separate section, which is then followed by a discussion on phenotypic switching at a cellular level.

##### Colony morphology switching in *Candida albicans*—initial discovery

**Figure 2.**
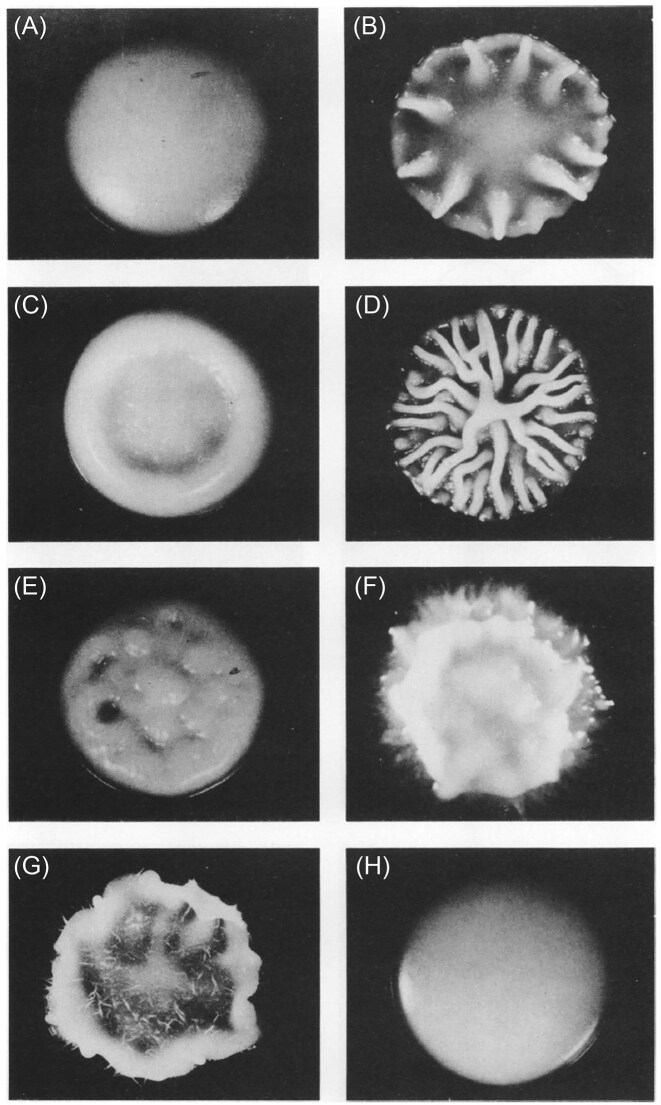
A prominent example of phenotypic switching in colony morphology phenotypes reported by Slutsky et al. (1985). The eight *Candida albicans* colony types illustrated include the original smooth white colony (A) and seven spontaneously sectoring phenotypes (designated as star (B), ring (C), irregular wrinkle (D), stipple (E), hat (F), fuzzy (G), and r-smooth (H)) that differ in the degree of cell polarization and cell separation/attachment. Used with permission from Slutsky et al. (1985).

The first prominent example of phenotypic switching is in colony morphology switching in *Candida albicans*, first discovered nearly 40 years ago (Fig. 2) (Slutsky et al. 1985). The authors documented colony morphologies switching between at least eight colony types, including the original smooth colony and seven spontaneously sectoring phenotypes (designated as star, ring, irregular wrinkle, hat, stipple, fuzzy, and r-smooth; Fig. 2) at a frequency of ∼1/10 000 divisions (Slutsky et al. 1985). A distinctive feature of colony morphology heterogeneity, now recognized also in other species, is that switching between individual colony phenotypes can be reversible (Slutsky et al. 1985, Jain et al. 2008). The ability to switch morphologies is strain-specific, and presumably affected by strain genetic background (Soll 2014) but was seen in laboratory strains as well as in isolates from patients with candidiasis. Each of the two major fungal morphological states, yeast and hyphae exhibit intrinsic variability in morphology. Technically, it is simpler to identify switches in phenotypes from the yeast form, as altered properties are readily observed as deviations from smooth round colonies to form sectoring patterns.

##### White/opaque switching in *Candida albicans*—a connection between colony morphology and cellular morphology

Typically, *Candida albicans* yeasts form white, smooth colonies. The white/opaque switch is a cell morphology shift first identified in the WO-1 strain, which was originally isolated from the blood of a patient suffering from systemic candidiasis (Slutsky et al. 1987). [L436] The “white” morphological type cells formed colonies that were white and hemispherical. By contrast, the “opaque” cell type formed colonies that were larger, flatter, and opaque, or gray (Slutsky et al. 1987). White/opaque switching was detected as phenotypic switching between these two major, stable, high-frequency colony phenotypes (Slutsky et al. 1987); much later, Miller and Johnson (2002) discovered that the white/opaque switch occurs at much higher frequencies in cells homozygous for the mating type-like locus (*MTL*) (Miller and Johnson 2002), and that the WO-1 strain was hemizygous for the *MTLa1* gene. We now understand that the white/opaque switch usually is a prerequisite for mating between *Candida albicans* isolates of opposite mating types. Of note, *Candida albicans* is diploid and has no known meiosis, and thus mating between isolates generally yields tetraploid mating products that undergo occasional recombination events (Wang et al. 2018) and a process of “concerted chromosome loss” to return to a diploid or near-diploid aneuploid state (Bennett and Johnson 2003).

##### Effect of phenotypic switching on infections associated with *Candida albicans*

Opaque cells favor lower temperatures and are more invasive, making them effective in cutaneous infections; at body temperature (37°C), opaque cells switch to the white state, which is significantly more virulent in the bloodstream and other infection models (Kvaal et al. 1999). Cells from opaque colony sectors are larger, and more oblong in shape relative to “white” cells and are characterized by a rough or “pimpled” surface that is evident in scanning electron microscopy images. Furthermore, the surfaces/cell walls of opaque cells have opaque-specific antigens as well as one or more surface antigens thought to be hyphae-specific (Anderson and Soll 1987). The transcriptional program expressed upon W/O switching includes genes encoded by critical *Candida albicans* virulence factors including proteins important for adhesion to host tissues, hyphae formation, and pathogen recognition and neutralization by the immune system (Jain et al. 2008). For example, human neutrophils selectively phagocytose and kill white cells when exposed to both cell types (Sasse et al. 2013).

More recently, a third type of morphology, gray cells, was found in *Candida albicans* passaged through the mammalian gut (Pande et al. 2013). Gray cells exhibited elongated morphology and formed darker and flattened colonies, in contrast to the original white cells (Pande et al. 2013). Cells in the gray state can arise from either white or opaque cells and possess high infectivity potential in cutaneous infections. Gray cells also are associated with a transcriptional program that is distinct from the transcriptomes of white and opaque yeast forms (Pande et al. 2013, Tao et al. 2014), supporting the idea that gray cells are a unique alternative phenotypic state.

##### Phenotypic switching in species other than *Candida albicans* and its relevance to virulence

Based on the paradigm of the white–gray–opaque switching system in *Candida albicans*, similar forms of phenotypic heterogeneity likely play an important role during infections caused by other fungal species. Indeed, phenotypic variants that cause colony sectoring have been characterized in *Nakaseomyces glabrata* (formerly *Candida glabrata*), *Candida lusitaniae, Candida parapsilosis, Candida tropicalis*, and the basidiomycetous yeasts *Cryptococcus gattii* and *Cryptococcus neoformans* (Goldman et al. 1998, Lachke et al. 2002, Laffey and Butler 2005, Jain et al. 2006, Miller et al. 2006, Porman et al. 2011). [L477]

Goldman et al. (1998) described three colony types, with significant differences in virulence and immune responses in the rat and mouse models of *Cryptococcus neoformans* infection. One of the three types had a significantly larger capsule, which may partly explain differences in pathogenesis, as it elicited the least inflammation (Goldman et al. 1998). Interestingly, cells from colony types that exhibited the highest virulence in the murine tail vein injection model triggered the least inflammation in the rat model of lung infection (Goldman et al. 1998), suggesting that switching to cell types that elicit minimal inflammation contributes to the persistence of infection (Goldman et al. 1998). Importantly, the switch between phenotypic variants of *Cryptococcus neoformans* has been detected also *in vivo*, and antifungal treatment may select for specific variants relative to others, despite the apparent lack of differences in *in vitro* drug susceptibility measured for each of the phenotypic variants (Fries et al. 2001, 2005). Overall, it appears that *Cryptococcus neoformans* undergoes phenotypic switching in the host, resulting in phenotypic variants with different selective advantages that may enable them to persist during infection under specific host conditions.

#### Impact of cell size heterogeneity on yeast infections

Dramatic differences in cell size are a prominent type of heterogeneity in genetically identical yeast cells. *Candida albicans, Candida dubliniensis*, and *Candida tropicalis* all can form enlarged “goliath” cells when grown *in vitro* under zinc-limiting conditions (Malavia et al. 2017). During commensal colonization and pathogenic activity, *Candida albicans* resides in host niches deprived of zinc, suggesting that goliath cells may be formed during infection, although there is no direct evidence of this. In addition, goliath cells exhibit increased adhesion *in vitro*, further suggesting a possible role in pathogenicity (Malavia et al. 2017). Enlarged cells were also observed in *Candida auris* when cultures were treated with the genotoxic agent methyl methanesulfonate; whether those cells represent a morphotype similar to goliath cells remains unclear (Bravo Ruiz et al. 2020). Considerable progress in studies on yeast size heterogeneity and its impact on infection has been made based on enlarged “titan” cell studies in the *Cryptococcus neoformans/gattii* species complex, as discussed next.

##### Cell size heterogeneity in the *Cryptococcus neoformans/Cryptococcus gattii* species complex

Pathogenic basidiomycetous yeasts of the *Cryptococcus neoformans/Cryptococcus gattii* species complex are responsible for HIV-associated cryptococcal meningitis in hundreds of thousands of people per year (Rajasingham et al. 2017). As with the dimorphic pathogens, yeast cells cause cryptococcosis, although yeast is also the predominant environmental form, and the hyphal form is only restricted to mating that presumably occurs in the environment (Wang et al. 2012).

Cryptococcal yeast cell size exhibits strain-specific and species-specific complex heterogeneity both *in vitro* and *in vivo* (Feldmesser et al. 2001, Dambuza et al. 2018). For example, some *Cryptococcus neoformans/Cryptococcus gattii* species complex strains form enlarged “titan” cells, defined as >10 µm in diameter with thick cell walls, a single large vacuole, and >1 N DNA content within a single nucleus (Okagaki et al. 2010, Zaragoza et al. 2010, Li and Nielsen 2017). Titan cells are morphologically different from enlarged cells in *Candida* species and may represent a distinct morphotype unique to the *Cryptococcus neoformans/Cryptococcus gattii* species complex (Dylag et al. 2020).

External signals that stimulate the formation of titan cells include CO_2_, hypoxia, fetal bovine serum, phospholipids, bacterial cell wall components, and pH (Dambuza et al. 2018, Hommel et al. 2018, Trevijano-Contador et al. 2018, Dylag et al. 2022, Saidykhan et al. 2022). *In vitro* conditions that restrict proliferation in the presence of 5% CO_2_ are sufficient to induce titan cell formation (Dylag et al. 2022, Saidykhan et al. 2022). Other host-derived factors that modulate titan formation during infection remain to be established. Titan cell formation requires cell cycle and mating pathways that trigger CAMP/protein kinase A signaling (Okagaki et al. 2010, 2011, Choi et al. 2012, Trevijano-Contador et al. 2018, Altamirano et al. 2021, Cao et al. 2022), among other pathways that differ, depending on the background of nuclear-encoded, but not mitochondrial-encoded genes (Saidykhan et al. 2022).

Originally identified in a mouse lung infection model, titan cells are thought to promote fungal survival in the host, as the large cells are resistant to phagocytosis (Zaragoza et al. 2010, Crabtree et al. 2012, Okagaki and Nielsen 2012, Zaragoza and Nielsen 2013, Reuwsaat et al. 2021). Titan cell progeny can be aneuploid and exhibit elevated resistance to environmental stresses, including antifungal drug exposure (Gerstein et al. 2015). Titan cell wall and capsule composition differs from typical yeast cells (Mukaremera et al. 2018), which may promote enhanced fungal dissemination within the host (Crabtree et al. 2012, Gerstein et al. 2015). However, more specific roles of titan cells in pathogenesis remain to be discovered (Dambuza et al. 2018, Hommel et al. 2018, Trevijano-Contador et al. 2018).

Titan cells are a type of phenotypic heterogeneity, in that only some cells within a population form titan cells, both *in vitro* and *in vivo* (Zaragoza and Nielsen 2013). Why only some cells transition to the titan state and whether size heterogeneity is important for pathogenicity are not known. Some *Cryptococcus neoformans* clinical isolates produce few if any titan cells in *in vitro* experiments (Hommel et al. 2018). Furthermore, in two studies, mutants that produce *more* titan cells are *attenuated* for virulence in the mouse model of cryptococcosis (Masso-Silva et al. 2018, Reuwsaat et al. 2021), presumably because of the inability of these large cells to cross biological barriers. These studies suggest that a balance between cell types of different sizes may be critical for cryptococcosis and that titan cell formation may not be required for all cryptococcal infections.

*Cryptococcus neoformans* also can form small “seed cells” morphotypes both *in vitro* and *in vivo* (Feldmesser et al. 2001, Dambuza et al. 2018, Denham et al. 2022, Freitas et al. 2022). Seed cells are induced during infection and contribute to dissemination out of the lung (Denham et al. 2022). *In vitro* induction of seed cells requires elevated phosphate levels. It is thought that the progression of cryptococcosis from the lungs to the central nervous system (CNS) is linked to dynamic changes in phosphate availability; lung phosphate levels are initially low (and can promote early titan cell formation) and increase with time due to acidification of the lung environment around *Cryptococcus neoformans* cells, which promotes a predominance of seed cells that facilitate dissemination into the bloodstream and CNS (Denham et al. 2022).

Strikingly, while *Cryptococcus neoformans* cells associated with lungs and other organs exhibit high phenotypic heterogeneity of yeast cell size, cells that make their way into the brain, the “final destination” of the pathogen, are relatively small and much less variable in size (Denham et al. 2018). This suggests that size heterogeneity is, at least in part, a response to host environmental conditions. It is also consistent with the idea that phenotypic heterogeneity is a complex trait that integrates environmental inputs with diverse genetic background effects.

#### Conidial and spore heterogeneity in filamentous fungi

Another morphological heterogeneity relevant to pathogenicity is associated with the sexual versus asexual formation of spores or conidia, respectively. Both are infectious propagules whose rate of germination and cell wall composition significantly influence the progression of infection.

In *A. fumigatus*, conidia vary in germination rate, size, and capacity to infect the *Galleria mellonella* host, and this variation is dependent upon specific environmental conditions (Earl Kang et al. 2021). Specifically, conditions that promoted faster germination yield less variability in germination time, suggesting that the sporulation environment drives variation in germination characteristics, which may provide a bet-hedging strategy to ensure progeny survival in a broad range of environments (Earl Kang et al. 2021).

*Aspergillus fumigatus* conidia also exhibit cell wall composition heterogeneity, which can be observed by staining mannose, chitin, and *N*-acetylgalactosamine cell wall polysaccharides (Bleichrodt et al. 2020). Different levels of heterogeneity were detected, depending on the measured component of the cell wall and the developmental stage of the conidia. Importantly, cell wall composition heterogeneity conferred fitness in the presence of caspofungin, providing a link between phenotypic heterogeneity and an antifungal drug response (Bleichrodt et al. 2020). The deletion SrgA, a Rab GTPase, dramatically increased the phenotypic heterogeneity detected as colony sectoring, attenuated stress responses, and reduced virulence in *G. mellonella* (Powers-Fletcher et al. 2013). However, whether the phenotypic heterogeneity observed is associated with non-genetic variation or an elevated mutation rate remains to be determined (Powers-Fletcher et al. 2013).

Spore heterogeneity also was linked to virulence in, *Mucor circinelloides*, a zygomycete responsible for life-threatening mucormycoses (Chayakulkeeree et al. 2006, Li et al. 2011). *Mucor circinelloides f. lusitanicus (−)* mating-type isolates produce larger and more heterogeneously sized asexual sporangiospores that are more virulent in *G. mellonella* (Li et al. 2011). Larger spores were multinucleate, and germinated faster in media as well as in macrophages, leading to macrophage death. By contrast, smaller spores had delayed germination in media and remained dormant after phagocytosis, suggesting that the rapid germination of larger spores is critical for increased virulence. However, it remains unclear whether heterogeneity of spore size affects *Mucor* infections *i n vivo* (Li et al. 2011). Thus, the heterogeneity of spores and/or conidia appears to be a common mechanism for fungi to generate diversity in response to adverse environments, including those presented by the host immune responses.

### Relationship between phenotypic heterogeneity and antifungal drug responses

Fungal cell growth in the presence of an antifungal drug can be driven by *bona fide* resistance to the drug or from antifungal drug tolerance (Perlin et al. 2017, Berman and Krysan 2020). Resistance mechanisms are usually due to genetic changes that directly reduce drug susceptibility either by reducing the affinity of the drug target for the drug, increasing the amount of the target, or by decreasing the amount of drug in the cell (Perlin et al. 2017); a classic example is the upregulation of efflux pump expression via increased transcription or increased gene copy number. Resistant isolates pose a significant therapeutic challenge because they render specific drugs or drug classes therapeutically ineffective. The few types of genetic changes that confer *bona fide* resistance are well defined and thus can be identified with molecular diagnostics that then recommend the use of alternative drugs, often from different drug classes (Lee et al. 2021). The limited number of available antifungal drug classes is another constraint that challenges our ability to clear infections caused by resistant fungal isolates.

The basis for antifungal drug tolerance is usually phenotypic rather than genetic and is more subtle than the origin of drug resistance. Subpopulations of clonal cells exhibit slow growth, and this growth is often heterogeneous in the population, with some cells growing, while others are inhibited. Tolerance requires the activity of multiple stress response pathway hubs and is likely a quantitative trait affected by the diverse genetic backgrounds of clinical isolates (Berman and Krysan 2020, Lee et al. 2021).

Addressing tolerance will require an understanding of how genetic background differences contribute to phenotypic (and metabolic) heterogeneity across cells in a clonal population, and how cellular stress responses are integrated into the process. Does the outcome of these processes cause changes in drug efflux or drug–target interactions (to a lesser degree than in drug-resistant mutants) or do mechanisms of cellular resilience allow alternative mechanisms to continue cell growth despite the presence of drug stress?

In the following sections, we discuss phenotypic heterogeneity in response to antifungals largely based on studies that investigated the effect of fluconazole, an azole drug, on *Candida albicans* and *Cryptococcus neoformans*. For information including other drugs and other fungal species, we refer the reader to reviews focused on the subject of antifungal resistance (Perlin et al. 2017, Berman and Krysan 2020).

#### Definitions of drug resistance, tolerance, heteroresistance, and persistence

Antifungal drug resistance, like resistance in bacteria and other eukaryotic pathogens, is generally due to mutations that alter the drug–target interaction, either directly, by modifying the drug binding site in the target, or indirectly, by altering the stoichiometry of the drug relative to the target. The latter can occur via the expression of excess target or by effluxing more of the drug. Resistant cells grow relatively well in the presence of the drug, with all cells in the population usually exhibiting a similar rate of growth in the presence of drug concentrations considered inhibitory for most isolates of the species.

The term “tolerance” is more ambiguous and differs for different organisms and their responses to different drug classes. Antibacterial tolerance definitions emphasize the ability of all cells to survive transient exposure to a bacteriocidal drug and may be due to genetic or non-genetic changes (Brauner et al. 2016). Bacterial tolerance can be quantified by estimating the time required for a cidal drug to kill 99% of cells in a given population [L671].

Antifungal drug tolerance has different properties depending on the organism, the drug, and the type of growth being measured. Studies with *Candida albicans* and the fungistatic azole antifungals describe fungal tolerance as the slow growth of a subpopulation of cells at or above the MIC determined for the population. Colonies initiated by “tolerant” *Candida albicans* cells take more time to appear (usually 48 h vs the 24 h time point recommended for determining resistance) and thus are smaller than colonies formed by resistant isolates (Rosenberg et al. 2018). Thus, in clinical assays that measure susceptibility at 24 h, a subpopulation that is reflective of tolerance often tests as susceptible. The appearance of tolerant subpopulations after 48 h is akin to the “trailing growth” noted in several clinical studies, and high tolerance/trailing growth appears to be associated with more persistent candidal infections (Revankar et al. 1998).

Heteroresistance as described in bacteria is a phenomenon where an isogenic population exhibits a range of drug susceptibilities among individual cells (El-Halfawy and Valvano 2015, Andersson et al. 2019). Furthermore, bacterial heteroresistance is attributed to a wide variety of genetic, epigenetic, and nongenetic mechanisms (El-Halfawy and Valvano 2015).

**Figure 3.**
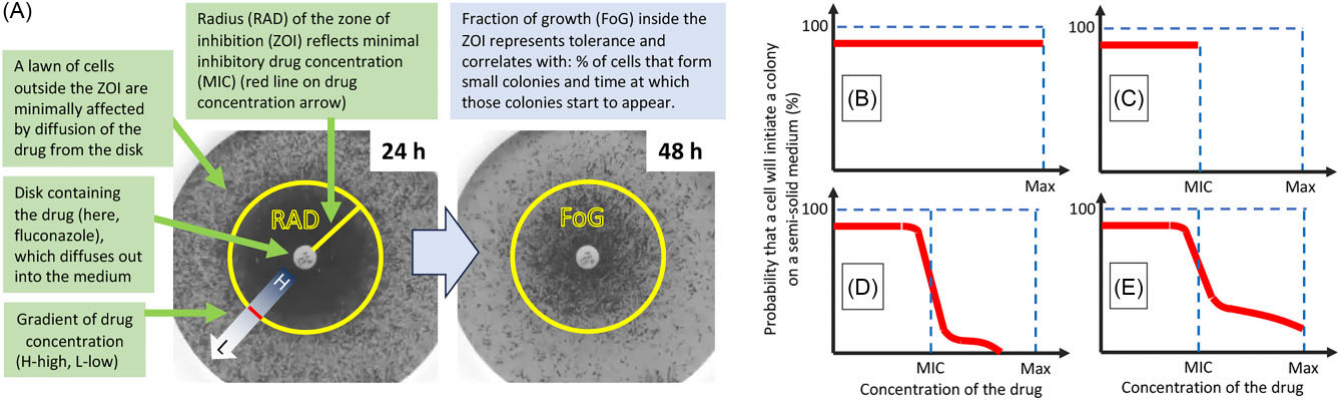
Disk diffusion assay is a useful method to assess antifungal drug resistance and tolerance. (A) Disk diffusion assay showing same plate photographed at 24 and 48 h after spreading *Candida albicans* cells on the plate. (B) A population in which *all* cells are resistant to the drug up to the maximum concentration tested. (C) A population in which *all* cells are susceptible to a specific drug concentration (MIC). (D, E) Two examples of populations with different tolerance levels relatively low (D) or high (E), with a proportion of the population forming colonies at supra-MIC concentrations. The shape of the curve is population-specific. Tolerance levels depend on the proportion of cells that initiate growth above the MIC level and the time it takes for colonies to start appearing (Rosenberg et al. 2018).

In *N. glabrata* (formerly *Candida glabrata*) and *Candida albicans*, heteroresistance has been described based on the bacterial paradigm (Ben-Ami et al. 2016, Gautier et al. 2024). Gautier et al. (2024) exposed *Candida albicans* to a disk diffusion assay (Fig. 3A) and isolated colonies within the zone of inhibition that exhibited subsequent resistance at levels 1.2- to 256-fold higher than the parent population, in an isolate-specific manner (Gautier et al. 2024). They defined heteroresistant colonies as those that exhibited at least 10-fold higher MIC_50_ than the parent population (Gautier et al. 2024).

In *Cryptococcus neoformans*, the term “heteroresistance” was defined as an intrinsic ability to become fluconazole-resistant when exposed to inhibitory concentrations of fluconazole (Sionov et al. 2009, 2010, 2013). This acquisition of resistance (tested as an ability to grow at a concentration that is twice as much as the original MIC) is often due to acquired aneuploidy (Sionov et al. 2009, 2010, 2013, Stone et al. 2019). Extra copies of specific genes that contribute to fluconazole heteroresistance in *Cryptococcus neoformans* are well established and include *ERG11*, which encodes the azole target, and AFR1, which encodes an efflux pump (Sionov et al. 2010). Aneuploidy of a chromosome that carries one or more of these genes often provides a selective growth advantage in an antifungal drug, a feature seen in *Candida albicans* as well (Selmecki et al. 2009, 2010, Kwon-Chung and Chang 2012).

Antibacterial persistence refers to the ability of a very small fraction of cells (usually <1%) to survive transient exposure to a bacteriocidal drug (Brauner et al. 2016). The term “persistence” also was used to refer to a small subpopulation of non-growing cells found in *Candida* spp. biofilms upon exposure to amphotericin B, a drug considered fungicidal (Wuyts et al. 2018). However, the initial reports of *Candida albicans* persister cells in biofilms may be due to an artifact (LaFleur et al. 2006, Denega et al. 2019). Specifically, the growth conditions used caused a small fraction of cells to be deposited at the border of the air and liquid phases during incubation. When the protocol was modified to eliminate this accidental cellular deposition, persister cells were not detected. This suggests that these deposited cells might have “escaped” the drug rather than having been true persisters. Furthermore, an alternative protocol of *Candida albicans* biofilm formation yielded few, if any, persister cells, with the authors suggesting that this very small fraction of “persisters” might be due to experimental error (Al-Dhaheri and Douglas 2008). Thus, while evidence for persister cells in the biofilms of *Candida albicans* and related species is weak in these *in vitro* studies, the existence of persister cells during infection cannot be excluded (Denega et al. 2019).

#### Phenotypic heterogeneity in response to fluconazole

Azoles inhibit lanosterol 14α-demethylase, Erg11p, an essential enzyme for the biosynthesis of ergosterol, an important sterol present in fungal cell membranes (Zhang et al. 2010). Fluconazole, similar to other azoles, inhibits growth, rather than killing the majority of cells in the population, thereby providing an opportunity for resistance to emerge. Accordingly, strains that exhibit higher tolerance levels also produce more dividing cells. Because higher tolerance levels yield a larger population size, they may promote the acquisition of resistant mutations. However, the question of whether tolerant isolates always give rise to more resistant progeny may not be this simple and deserves further study.

Disk diffusion assays, as shown in recent studies involving *Candida albicans*, are useful for measuring antifungal drug resistance and tolerance (Fig. 3) (Gerstein et al. 2016). Let us consider the probability that a cell will initiate a colony on a semi-solid medium containing a gradient of the drug. For a strain that is resistant to the maximum achievable concentration of the drug, this probability is nearly 100%, along the entire concentration gradient (Fig. 3B). For a *bona fide* resistant strain, characterized by the MIC_50_ that is less than the maximum achievable drug concentration, this probability is nearly 100% at concentrations up to the MIC_50_ for that strain. If such a strain does not exhibit phenotypic heterogeneity, essentially 0% of cells should form colonies at inhibitory concentrations of the drug (above the MIC_50_) (Fig. 3C). However, many isolates of *Candida albicans* exhibit tolerance to fluconazole. The level of fluconazole tolerance in *Candida albicans* is measured at 48 h as the fraction of growth at supra-MIC drug concentrations, relative to growth without drug, whereas the resistance level is measured at 24 h as radius of the zone of inhibition (Fig. 3). The difference in intrinsic tolerance levels between different isolates is reproducible for that strain, and is assumed to be due to differences in genetic backgrounds (Rosenberg et al. 2018). Thus, the level of tolerance is a heritable feature characteristic of a specific isolate (Rosenberg et al. 2018) that is dependent upon subpopulation growth. The degree of growth of a tolerant subpopulation is associated with the time when tolerant colonies become evident on the plate, and the size oong periods without drug selectiof the subpopulation of tolerant cells (the proportion of cells that form “tolerant” colonies; Rosenberg et al. 2018) (Fig. 3). This intrinsic tolerance level of a given *Candida albicans* strain is assumed to be dependent upon multiple genes and stress pathways. Indeed, a range of adjuvant drugs can reduce tolerance and render fluconazole fungicidal, rather than fungistatic (Rosenberg et al. 2018). This implies that the adjuvants inhibit pathways that facilitate cell survival in the drug. The fact that different adjuvant drugs affect known stress response proteins and pathways, including Hsp90, calcineurin, Pkc1, mTOR, and sphingolipid biosynthesis, implies that tolerance requires many functional stress response pathways. Furthermore, each of these adjuvant drugs is cidal in combination with fluconazole, implying that either all the affected pathways are necessary for tolerance (and survival in fluconazole) or, alternatively, extensive cross-talk exists between the many stress pathways (Rosenberg et al. 2018). Either way, the elimination of any one of these stress pathways destroys cellular resilience in the face of drug stress. How this clear dependence on so many stress pathways is related to the phenotypic heterogeneity associated with antifungal drug tolerance remains to be determined.

Tolerance to fluconazole in *Candida albicans* can also be estimated using liquid assays, usually broth microdilution assays, as “supra-MIC growth” (SMG) (Rosenberg et al. 2018). SMG is measured as the growth at 48 h relative to growth at 24 h, at concentrations above the MIC_50_. The degree of correlation for tolerance levels differs between agar-based assays, which assess the growth of individual colonies within the zone of inhibition, and liquid assays, where competition for nutrients and relative growth rates of different subpopulations contributes to the average population density. Nonetheless, SMG levels are also generally heritable and represent a different way of measuring intrinsic antifungal drug tolerance.

##### Evolution of tolerance and resistance to fluconazole

Drug exposure can induce genetic changes, via both recombination and chromosome missegregation that yields new clones characterized by genetic or genomic changes (discussed later). Some of these genetic changes can drive the emergence of higher levels of acquired antifungal drug tolerance or resistance (Harrison et al. 2014). Increased growth in drug could also arise via changes in epigenetic features such as shifts between euchromatin and heterochromatin states (Torres-Garcia et al. 2020) or the induction of different protein or cytoplasmic states (e.g. via prions or the change in cytoplasmic condensate states; Halfmann et al. 2010, Hyman et al. 2014). When such states confer the ability to grow despite the presence of an inhibitory antifungal drug, we call them “acquired antifungal drug tolerance” or “acquired antifungal drug resistance.” Such states exhibit different levels of stability, with some being lost readily in the absence of drug selection, while others are retained for long periods without drug selection.

Acquired tolerance or resistance is often associated with the acquisition of specific aneuploid chromosomes (Yang et al. 2023). This “acquired tolerance” or resistance (referred to as heteroresistance in *Cryptococcus neoformans*) is lost when the aneuploid chromosome is lost and thus is semi-stable. It is important to note that exposure to stresses (e.g. heat shock; Bouchonville et al. 2009) and antifungal drugs can promote nuclear and chromosome segregation defects that result in altered cell cycle processes that result in ploidy shifts and aneuploidy in both *Candida albicans* and *Cryptococcus neoformans* (Harrison et al. 2014, Altamirano et al. 2017). Drug exposure also appears to promote recombination, which gives rise to loss of heterozygosity (LOH) and copy number variations (CNVs) that involve shorter tracts of genomic DNA in *Candida albicans* (Todd and Selmecki 2020).

In general, specific aneuploidies, LOHs, or CNVs are recurrently selected because they provide a selective advantage for the cells that carry them (e.g. Ford et al. 2015, Todd and Selmecki 2020). For example, acquired tolerance in *Candida albicans* cells exposed to fluconazole is often associated with the acquisition of altered numbers of all or part of chrR (Kukurudz et al. 2022, Todd et al. 2023, Yang et al. 2023). Similarly, *Cryptococcus neoformans* responds to fluconazole heterogeneously, with several distinct aneuploid patterns being selected; this suggests that aneuploidy arises randomly among individual cells (Sionov et al. 2010, 2013), and that specific sets of aneuploids provide a selective advantage for growth in the presence of the drug. The exact mechanism of aneuploidy induced by fluconazole remains elusive and may involve several possible scenarios, including chromosomal nondisjunction, endoduplication, or other, yet to be identified phenomena (Harrison et al. 2014, Altamirano et al. 2017, Chang et al. 2018).

In experimental evolution experiments with *Candida albicans* clinical isolates having different intrinsic tolerance levels, exposure to fluconazole induced *acquired tolerance* relatively rapidly (within 24 h) at all drug concentrations above the MIC. Furthermore, this acquired tolerance was associated with a similar set of recurrent aneuploidies, irrespective of the supra-MIC drug dose. This indicates that the acquisition of tolerance and its selection occur via mechanisms that operate once drug–target interactions are saturated, and suggests that the mechanisms are independent of drug concentration.

By contrast, acquired resistance (in which all cells grow above the original MIC_50_ level within 24 h) emerged only after 10–15 passages, appeared primarily at drug concentrations *below* the MIC (Yang et al. 2023) and the majority of these resistant clones were not aneuploid. However, there were rare resistant isolates that acquired aneuploid chromosomes. Importantly, the recurrent aneuploid chromosomes in the resistant adapters were different from those in adapters with acquired tolerance (Yang et al. 2023). This also supports the idea that tolerant and resistant cells respond to drugs via different mechanisms, with selection favoring extra copies of genes on different chromosomes.

Interestingly, external conditions that promote more robust *in vitro* growth of *Cryptococcus neoformans* (e.g. rich medium, or 30°C) decrease the degree of drug tolerance and resistance, relative to conditions promoting slow proliferation (25°C, minimal media) (Altamirano et al. 2018). Similarly, temperature and type of growth medium influence levels of tolerance to fluconazole in *Candida albicans* (Yang et al. 2023). The relationship between temperature and tolerance levels is isolate-dependent; ∼70% of 133 tested clinical isolates that were not tolerant at 30°C exhibited temperature-enhanced tolerance at 37°C and 39°C (Yang et al. 2023). The frequency of appearance of heteroresistance in *Candida albicans* also differs between strains (Gautier et al. 2024). Thus, many factors affect the proportion of colonies that grow in the presence of drug; these include temperature and nutritional factors, as well as genetic background (Altamirano et al. 2018, Yang et al. 2023, Gautier et al. 2024).

In summary, *intrinsic* tolerance is due to differences in genetic backgrounds in the context of euploid (non-aneuploid) genomes, while rapidly *acquired* drug-induced tolerance is often driven by the acquisition of aneuploidy or CNVs (Yang et al. 2023, Todd et al. 2023). The *Candida albicans* studies point to different evolutionary trajectories for drug adaptation that depend upon the drug concentration relative to the MIC. While it remains unclear why lower concentrations of fluconazole favor acquired resistance while higher drug concentrations promote acquired tolerance, the acquired resistance appears in a step-wise manner, suggesting that the trajectory of acquired drug resistance may traverse multiple stages in which cells are not yet able to grow in supra-MIC drug concentrations.

## Conclusion

Phenotypic heterogeneity in response to antifungal drugs raises two major interdependent challenges: (i) it enables the pathogen to continue dividing (albeit slowly) in the presence of inhibitory drug concentrations, and thus for the infection to persist and (ii) it may increase the degree to which the pathogen can escape immune responses that normally neutralize the pathogen. Two main deterministic factors, cell cycle stage and replicative age of the fungal cells, contribute to phenotypic heterogeneity by influencing the abundance of cellular constituents and their activity in individual cells. Fungi also exhibit a spectrum of specialized morphologies and colony phenotypes that contribute to the phenotypic heterogeneity of the population. Gene expression noise provides yet another level of phenotypic heterogeneity. In addition, specific stress conditions, including those found within the host, result in increased levels of expression noise. A better understanding of the molecular mechanisms that govern phenotypic variability will be critical to improving antifungal therapies.

Several aspects of phenotypic heterogeneity in fungi remain largely unresolved. First, it will be necessary to dissect the contributions due to genetics from those due to dynamic physiological or metabolic fluxes within a single genetic background. The involvement of epigenetic changes, such as chromatin modifications or partially heritable prion-like effects, remains a gray area that requires further exploration. It is especially difficult to disentangle genetic and phenotypic consequences, given that they are so intertwined: genetic background will influence the range of potential phenotypic heterogeneity in response to different environmental signals and, conversely, phenotypic heterogeneity may lead to genetic changes; for instance, cells that are “old” have increased genomic instability and increased mutation rates. Furthermore, we do not understand how most stresses affect phenotypic heterogeneity. For example, do all stresses act through one or several common mechanisms and what are the molecular mechanisms of crosstalk that might link them?

Another aspect that remains unclear is whether phenotypic heterogeneity is “heritable.” Even though by definition phenotypic heterogeneity is non-genetic in nature, certain cellular characteristics contributing to phenotypic heterogeneity may propagate through generations via epigenetic modifications or cytoplasmic inheritance of organelles and molecules. It remains unclear which heterogeneous phenotypes can be passed on to daughter cells and propagated, and how much inheritance of non-genetic traits is maintained or lost. The degree to which phenotypic traits may be inherited or lost during host infections, and during interactions with immune cells, also is not known.

Many examples of phenotypic heterogeneity discussed here refer to a limited repertoire of alternative phenotypic states. However, intermediate phenotypic states might be selected under specific environmental conditions. For example, a transition from the white to opaque cell variant in *Candida albicans* may proceed as a gradual process associated with continual changes in gene expression profile, resulting in the formation of cellular phenotypes that are intermediate between the two polar states, white and opaque.

The role of phenotypic heterogeneity in the evolution of drug resistance could be a stepping stone toward resistance (Todd and Selmecki 2020), by analogy with bacterial tolerance, which can promote the appearance of resistance (Balaban et al. 2004). Certainly, increasing the population size should increase the likelihood that stable genetic resistance mechanisms could arise, but whether other mechanisms (e.g. increased genome instability) also play a role remains to be determined. This may require the analysis of single cells as they evolve within a population.

An important question is whether understanding phenotypic heterogeneity can improve therapeutic outcomes by reducing the frequency of treatment failure. One strategy could be to use two drugs with different modes of action (Vitale 2021). Alternatively, the addition of adjuvant drugs that alone have no antifungal activity but that potentiate the antifungal activity of common antifungal drugs could reduce the emergence of resistance (Bibi et al. 2021) simply by reducing the number of viable cells. The rising incidence of non-*albicans Candida* infections and other unusual yeast species with higher intrinsic and acquired resistance levels is an additional factor clinically important (Miceli et al. 2011, Bays et al. 2024). Dealing with these infections will require clearer insights into phenotypic heterogeneity in non-*albicans* species such as *Candida auris, Candida parapsilosis, Candida lusitaniae, Candida tropicalis*, and *Nakaseomyces glabrata*. Ultimately, studies that increase our understanding of, and our ability to manipulate, the mechanisms underlying phenotypic heterogeneity should drive the design of more effective antifungal treatment strategies.
